# A numerical investigation of mathematical modelling in 3D hexagonal porous prism on oil recovery using nanoflooding

**DOI:** 10.1016/j.heliyon.2023.e18676

**Published:** 2023-07-26

**Authors:** Mudasar Zafar, Hamzah Sakidin, Mikhail Sheremet, Iskandar Dzulkarnain, Roslinda Nazar, Abdullah Al-Yaari, Nur Asyatumaila Mohamad Asri, Mohd Zuki Salleh, Shazia Bashir

**Affiliations:** a Department of Fundamental and Applied Sciences, Universiti Teknologi PETRONAS, Bandar Seri Iskandar 32610, Perak, Malaysia; b Center for Research in Enhanced Oil Recovery, Universiti Teknologi PETRONAS, Bandar Seri Iskandar 32610, Perak, Malaysia; c Laboratory on Convective Heat and Mass Transfer, Tomsk State University, 634050, Tomsk, Russia; d Department of Petroleum Engineering, Universiti Teknologi PETRONAS, Bandar Seri Iskandar 32610, Perak, Malaysia; e Department of Mathematical Sciences, Faculty of Science & Technology, Universiti Kebangsaan Malaysia, 43600 UKM Bangi, Selangor, Malaysia; f Centre of Mathematical Sciences, Universiti Malaysia Pahang, 26300 UMP Gambang, Kuantan, Pahang, Malaysia; g Department of Physics and Applied Mathematics and Centre for Mathematical Sciences, Pakistan Institute of Engineering and Applied Sciences, Nilore 45650, Islamabad, Pakistan

**Keywords:** Cavity, EOR, Mathematical modelling, Nanoflooding, Porous media

## Abstract

The use of nanomaterials as a means of recovering heavy and light oil from petroleum reservoirs has increased over the preceding twenty years. Most researchers have found that injecting a nanoparticle dispersion (nanofluids) has led to good results and increased the amount of oil that can be recovered. In this research, we aim to imitate the three-dimensional hexagonal prism in the existence of SiO2 and Al2O3 nanoparticles for better oil recovery. Porosity (0.1≤φ≤0.4), mass flow rate (0.05mL/min≤Q≤0.05ml/min), nanoparticle concentration (0.01≤ψ≤0.04), and the effect of relative permeability (kr) on oil and water saturation in the presence of gravity under different time durations are all investigated. The result obtained for the model is verified with existing experimental data. The results indicated that the infulence of nanoparticle volume fraction (VF) is significant in enhancing the oil recovery rate. It is also observed that at low porosity values the oil recovery is maximum. The maximum oil recovery is attained at low values of mass flow rate in the 3D hexagonal prism in the presence of silicon and aluminium nanoparticles It is also observed that the use of SiO2 gives a better oil recovery rate than Al2O3. It is also observed that maximum oil recovery is obtained at 99% at a flow rate of 0.05 mL/min in the presence of silicon injection.

## 1 Introduction

Even though a lot of money has been spent on renewable energy, none of these options has made it easier to meet the growing demand for energy. As a result, oil is the dominant source of energy for the time being. As a result, it is critical to extract as much oil as possible from existing wells before moving on to more newly discovered reserves. Companies and governments desired to obtain more oil out of these reservoirs after the first and second rounds of oil recovery, so they employed enhanced oil recovery (EOR) to recover the trapped oil deposited in the reservoirs. When primary and secondary recovery procedures are not possible, EOR technologies are used to extract more crude oil from the ground's surface. Some methods involve exploiting oil field energy or injecting materials into reservoirs. Typically, primary and secondary recovery technologies can only extract 20–40% of the oil from a reservoir. Nevertheless, EOR can extract 30–60% of the oil from a reservoir [[1], [2], [3]]. It is also noted that the finding of new oil with maximum capacity has been very difficult in the last 10 years, and the demand for oil is increasing every day, so there is a need to invent new methods or use new technologies in EOR to extract full oil from the existing reservoirs.

The use of nanotechnology in EOR increases oil recovery because the incorporation of nanoparticles (NPs) in the flooding process significantly improves EOR by modifying wettability, fluid behavior, trapped oil mobility, rock fusion, and decreasing interfacial tension (IFT) [4]. Multiple studies have used nanomaterials to control mobility, and the results have been great in terms of lowering water cuts, improving sweep performance, and getting more oil out of the ground. Moreover, NPs do not degrade in oil and gas reservoirs with high salinities and temperatures. Research has also investigated using nanofluids made of surfactant solutions and NPs to improve oil recovery in challenging reservoir settings. Researchers have used nanomaterials to reduce the viscosity of oil, heavy oil, and semi-heavy oil [[5], [6], [7], [8], [9], [10], [11], [12], [13]]. Several studies have also found that NPs can change how moist rocks are and accelerate the rate at which fluids travel through rocks. Both of these are the main mechanisms that NPs at the micro- and nanoscale may enable [[14], [15], [16]]. By incorporating nanofluid, it is possible to extract more than 50% of the oil from oil reservoirs—a feat that is not possible using primary, secondary, or even some EOR recovery techniques [17].

In the past few years, scientists have tried out different ways to use nanoflooding to improve oil recovery. In these tests, the amount of oil recovered from porous medium was shown to be more altered by nanofluids with varied nanoparticles and different operation settings than by water flooding. Most of the time, tests such as rheology (which examines changes in viscosity), surface tension, wettability, and core flooding are used to assess how well the EOR process works. The use of nanofluids in EOR has been studied to see how different nanoparticles and their properties affect the amount of oil that can be retrieved from porous media [[18], [19], [20], [21], [22]]. These experiments make use of a wide variety of nanoparticles, including SiO2, TiO2, CuO, and Al2O3 [[23], [24], [25], [26]]. When using nanoparticles to boost oil recovery, it is critical to define and understand the nature of nanoparticle impact for producing EOR approaches via nano-flooding. Recent research has shown how EOR works in some cases. In these cases, porous media, oil, and nanofluids interact with each other. Unfortunately, there is currently a paucity of knowledge regarding EOR techniques utilizing nano-flooding. Recent studies have demonstrated that nanoparticles can speed up oil recovery in a variety of ways. They include reducing the interfacial pressure, closing pore channels, reducing surface tension, changing the rock's ability to absorb water, and improving the injectable fluid's transfer qualities [18,[27], [28], [29], [30]].

The majority of study on nano-flooding has been experimental, but scientists can advance the area and take a helpful step towards industrializing the procedure by using modelling. In this case, the model results can be a helpful addition to the experimental data. Although nanoflooding has been used in several experimental studies in the field of EOR, very few research projects have focused on the numerical and modelling aspects of this procedure. Nano-flooding modelling for EOR can be carried out by anticipating the flow transfer of nanoparticles in porous media. When modelling flow transfer using nanoparticles, it is common to consider both the surface forces of the nanoparticles and the surface of the rock in the porous medium that is present during flow transfer. The wettability change phenomenon of rock surfaces is connected to nanoparticle absorption in porous media. This phenomenon defines both the relative permeability and capillary pressure curves. As a result, the variation in capillary pressure and relative permeability of the phases that occurs as a direct result of the wettability of the surrounding environment is used to characterize and mimic the process [31].

Ju et al. [32] did a numerical analysis to find out how poly-silicon hydrophilic fatty acid nanoparticles (LHP) improve oil recovery by changing how much water can get into porous media. They did this to find out how LHP affect oil recovery. In this study, a two-phase mathematical model for a one-dimensional geometry is put forth. This model suggests the migration and absorption of LHP nanoparticles as well as the resulting alteration in the wettability of the reservoir's rock medium as mechanisms for the EOR process. Transfer equations for nanoparticles in porous media have been used in the development of this model. The accumulating rate of nanoparticles has also been considered in these transfer equations. This is due to the possibility of nanoparticles getting stuck in gaps and medium bottlenecks. The equations for the transport of nanoparticles in porous media, which are based on the original formulation by Liu and Civan [33], are expanded in the model proposed by Ju et al. [34]. It can be used to simulate the flow of micron-sized particles through porous material. This model calculates the change in oil recovery rate after injecting water-containing nanoparticles, as well as the change in relative and effective permeabilities of the water and oil phases. This model has also been used to estimate the success of the oil recovery process, as well as the distribution of nanoparticle concentrations, a decrease in porosity, and the absolute permeability of the medium due to nanoparticle absorption in cavities and bottlenecks in porous media. Despite the fact that nanoparticle deposition made the porous medium less porous, Ju et al. [34] discovered that using hydrophilic LHP nanoparticles considerably enhanced the oil recovery rate of the environment. Ju and Fan [32] confirmed the findings of this study in a separate study.

Based on the previously discussed research, injecting various nanofluids improves a variety of properties such as wettability, IFT reduction, rheology, mobility control, and more. It should be noted that, although certain mechanisms influence reservoir rock features, others influence and modify trapped oil characteristics. It should be emphasized that EOR is based on several mechanisms that will function to produce EOR. Injecting nanofluids into a porous medium containing petroleum product provides the foundation for several of these mechanisms, including the previously described ones. As a result, nanofluids and nanoparticles can be regarded as potential EOR process approaches.

In this study, we simulate a nanofluid-injected, increased oil recovery hexagonal prism cavity. In this research, a method known as finite volume is used to numerically solve the governing equations. Moreover, the oil recovery rate in a 3D hexagonal prism cavity hasn't been investigated in any of the earlier investigations. The cavity's shape is being researched because it closely resembles the actual characteristics of the heterogeneous porous reservoir. By examining the effects of various volume fractions and porosities, flow rates, and permeability at various times, the goal of this work is to compute the oil recovery rate using nanofluid injection. In the flooding process, silicon and aluminium nanoparticles are employed.

## 2 Mathematical modelling

The assumptions behind the mathematical model that simulates two-phase flow with dispersed nanoparticles in three dimesional hexagoanl prism to find oil recovery rate are as follows:

i. The flow considered to be as one dimensional.
ii. Reservoir rock assumed to be clean sandstone.
iii. Fluids are considered as incompressible.
iv. The fluid flow obeys Darcy’s Law
v. Fluid is Newtonian fluid.
vi. The chemical reaction between the nanoparticles is neglected.
vii. Fluid flow assumed to be isothermal.
viii. Initially viscosity and density of the water and oil are constant.

To get better results from a reservoir simulation, it is very important to make the correct flow geometry and include the physical properties. Table 1 lists the parameters (physical and chemical properties) that are used to construct the geometry of a reservoir for simulation.

**Table 1:** The Construction of 3D Hexagonal Prism for nanofluid injection.

	Explanation of Physical and Chemical Properties	Quantity	S.I unit
**Construction of hexagonal prism**	Base length of prism	0.30	m
	Height of the prism	0.12	
**Core Volume**	Volume of the porous core inlet section of nanofluid flow	0.049	$m^{3}$
**Inlet cross sectional area (A)**	Inlet cross sectional area of nanofluid flow	0.45	$m^{2}$
**Physical Properties of the model**	• Inlet fluid temperature $T_{in}$	300	K
	• Initial system temperature $T_{initial}$	275	
	• Initial Pressure $P_{initial}$	1 atm	
	• Fluid outlet pressure $P_{out}$	1 atm	
	• Initial saturation of the oil phase system $S_{w}^{0}$	0.10	
	• Nanofluid Inlet Flow $Q_{in}$	12	PV/year

In this flooding process, we investigated the silica and aluminium nanoparticles to find out the oil recovery rate, and the physical properties of these nanoparticles along with reservoir parameters are shown in Table 2.

**Table 2:** Physical and Chemical Properties of the nanoparticles and Reservoir [35].

	Explanation of Physical and Chemical Properties	Quantity	S.I unit
**SiO2**	• Nanoparticle density (p_p_ ).	2220	Kg/ $m^{3}$
	• The specific heat of the nanoparticles	745	J/Kg. K
	• Thermal conductivity coefficient of the nanoparticles $k_{p}$.	36	W/m. K
	• VF of the nanoparticles (phi)	0.01	–
	• Diameter of the nanoparticles $(d_{p}$).	40	nm
	• Molecular mass of the nanoparticles (MNp)	60	g/mol
**AL203**	• Nanoparticle density ($\rho_{p})$.	3970	Kg/ $m^{3}$
	• The specific heat of the nanoparticles	765	J/Kg. K
	• Thermal conductivity coefficient of the nanoparticles $k_{p}$.	40	W/m. K
	• VF of the nanoparticles (phi)	0.01	–
	• Diameter of the nanoparticles $(d_{p}$)	40	nm
	• Molecular mass of the nanoparticles (MNp)	101.96	g/mol
**Properties of Oil at 300K**	• Oil density $\rho_{o}$.	829	Kg/ $m^{3}$
	• Oil heat capacity $C_{0}$.	1670	J/Kg. K
	• Oil thermal conductivity $k_{0}$.	0.13	W/m. K
	• Oil viscosity $\mu_{o}$.	1.15 $\times 10^{2}$	Pa. s
**Properties of water at 300K**	• water density $\rho_{w}$.	990	Kg/ $m^{3}$
	• water heat capacity $C_{w}$.	4200	J/Kg. K
	• water thermal conductivity $k_{w}$.	0.6	W/m. K
	• water viscosity $\mu_{w}$.	$10^{-3}$	Pa. s
**Properties of the rock reservoir**	• Rock density	2714	Kg/ $m^{3}$
	• Mesh diameter $d_{g}$.	3	μm

### 2.1 Governing equations

**Fig. 1:**
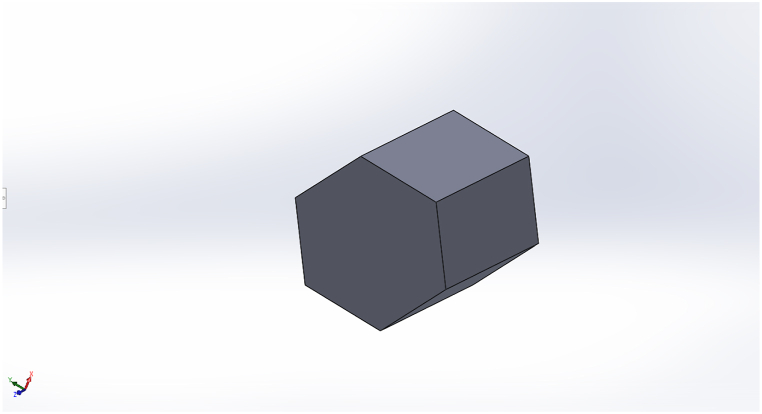
Geometry of the problem.

The following set of nonlinear partial differential (NPDEs) equations constitutes the two-phase math model for 3D hexagonal prism geometry: Fig. 1 depicts the problem's geometry. The Naiver-Stokes equations should be simplified in order to determine pressure and speed based on the flooding scenario. Inertia and force don't matter because oil reservoirs have modest oil flow rates. Because of the sluggish flow and low Reynolds number in a porous oil reservoir, Darcy equations can be used to calculate the velocity and pressure. Finding the amount of saturated oil and the amount of fluid used to flood is the goal of solving these equations.

(1) $$\frac{\partial \varphi \rho }{\partial t}+\frac{\partial \rho u}{\partial x}=0\text{,}$$

(2) $$u=\frac{k}{\mu }\;\frac{\partial p}{\partial x}$$

(3) $$\rho =s_{w}\rho_{w}+s_{o}\rho_{o}$$

(4) $$\frac{1}{\mu }=s_{water}\frac{k_{rw}}{\mu_{rw}}+s_{oil}\frac{k_{r0}}{\mu_{o}}$$

(5) $$\frac{\partial \varphi c_{w}}{\partial t}+\frac{\partial }{\partial x}\left(c_{w}u\right)=\frac{\partial }{\partial x}\left[.\left(D_{c}\frac{\partial }{\partial x}c_{w}\right)\right]$$

(6) $$c_{w}=s_{water}\rho_{water}$$

(7) $$D_{c}=\frac{k_{rw}}{\mu_{w}}+K\;\left(s_{water}-1\right)\;\frac{\partial p_{c}}{\partial s_{water}}$$

It is possible to calculate the capillary pressure using a logarithmic approach. We suppose the saturation equation is 0.15 since oil reserves typically have some water in them. We will determine the velocities, pressures, and saturations of oil and water employing Eqs. (1), (2), (3), (4), (5), (6), (7). Using nanofluids rather than water is one contemporary method to increase flooding's effectiveness. Also, when a nanofluid's inlet temperature is high, heat is transferred to the oil, reducing its viscosity and hastening recovery. Eq. (8) gives a definition for the mass transfer of the nanofluid.

(8) $$\frac{\partial \varphi S_{w}\psi_{w}}{\partial t}+u_{w}.\frac{\partial \psi_{w}}{\partial x}=\frac{\partial }{\partial x}.\left(\varphi S_{w}\psi_{w}D_{w}\frac{\partial \psi_{w}}{\partial x}\right)-R_{\iota }$$

The values of $R_{\iota }$ can be calculated by using Eq. (9).

(9) $$R_{\iota }=\frac{\partial \omega }{\partial t}+\frac{\partial \omega *}{\partial t}$$

The values of ω and ω* be considered using Eq. (10) [36,37].

(10) $$\frac{\partial \omega }{\partial t}=K_{d}\upsilon C,\frac{\partial \omega *}{\partial t}=K_{p}\upsilon C$$

Eqs. (11), (12), (13) is used to find the porosity, relative and absolute permeability which are given below,

(11) $$\varphi =\varphi_{initail}-\sum o_{i}^{*}+o_{i}$$

(12) $$K_{rw,P}=\left(1-\Psi_{s}\right)K_{rw}+\Psi_{S}K_{rw,C}$$

(13) $$K_{ro,P}=\left(1-\Psi_{s}\right)K_{ro}+\Psi_{S}K_{ro,C}$$

Where the terms $\Psi_{s}$, can be calculated by using Eq. (14),

(14) $$X_{s}=\frac{S_{RPt}}{S_{SC}}$$

The values of $S_{RPt}$ and $S_{SC}$ is determined by using Eq. (15), (16),

(15) $$S_{RPt}=\beta \sum o_{i}^{*}+o_{i}\;\frac{6}{d_{p}}$$

(16) $$S_{SC}=7000\varphi \sqrt{\frac{\varphi }{K}}$$

The I. Cs and B. Cs for hexagonal prisms are given below from Eqs. 17–24,

(17) $$When\;t=0,original\;saturation\;of\;water\;is\;zero\;i.e.,s_{w}^{0}=0$$

(18) −n.ρu=0

(19) −n.q=0

(20) $$\rho u=\left({s_{w}\rho }_{w}+s_{o}\rho_{o}\right)U$$

(21) $$-n.D_{C}\nabla c_{w}=0$$

(22) $$t=0,s_{w}=0.15$$

(23) $$t=0,\left\{ \begin{array}{c} \Psi =0\\ \Psi =\Psi_{i} \end{array}\right.$$

(24) $$t=0,\left\{ \begin{array}{c} \omega =0\\ \omega_{i}=0 \end{array}\right.$$

### 2.2 Grid indecency

Grid independence analysis is essential for checking the accuracy and reliability of simulation results and validating the numerical models that are used in simulations. Throughout the simulation analysis procedure, the most cell-dense mesh was chosen in this study. We solved our model on eight different grids to validate the oil recovery parameters over time. Table 3 summarizes the results of all grid analyses.

**Table 3:** Grid Independency analysis for flooding process.

Mesh number	1	2	3	4	5	6	7
**Cell size**	25	250	2456	4802	13403	30251	325230

**Fig. 2:**
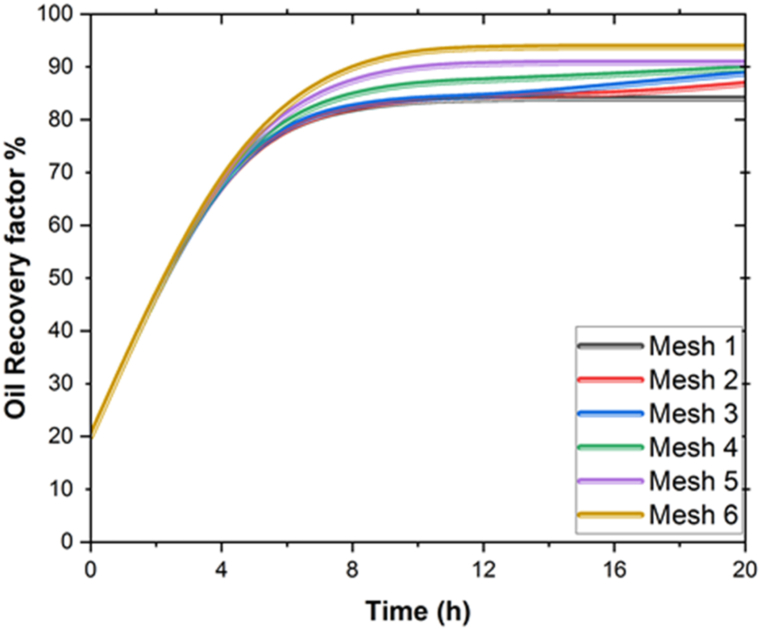
Analysis of Mesh on simulations.

Fig. 2 depicts the results of the grid's reliance on geometry. Grids 5, and 6, and 7 are, as can be seen, comparable to each other. This means that the mesh size has no effect on the model. The result is that grid number 7, with 325230 nodes, is the best mesh to use.

### 2.3 Validations

The veracity of the model can be assessed by contrasting its predictions with the findings of an experiment [35]. In this research, nanoparticles of SiO2 and Al2O3 were applied to a porous hexagonal prism to increase oil outflow. In Table 4, we can see the experimental parameters and rock core properties.

**Table 4:** The physical appearance of the core plugs [35].

Properties	Choice with SI
Diameter (D)	4.15 cm
Length(l)	5.78 cm
Permeability(K)	110.4 mD
Porosity (φ)	17.5%

**Fig. 3:**
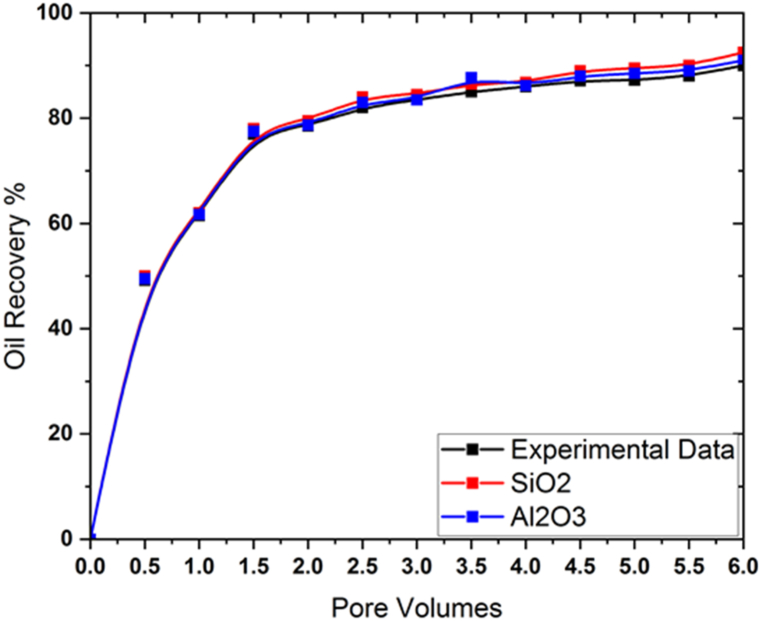
Verification of the results with experimental data [35].

Fig. 3 shows a graph comparing experimental data with model predictions. Fig. 3 shows a good agreement between the experimental data and the models.

## 3 Result and discussion

**Fig. 4:**
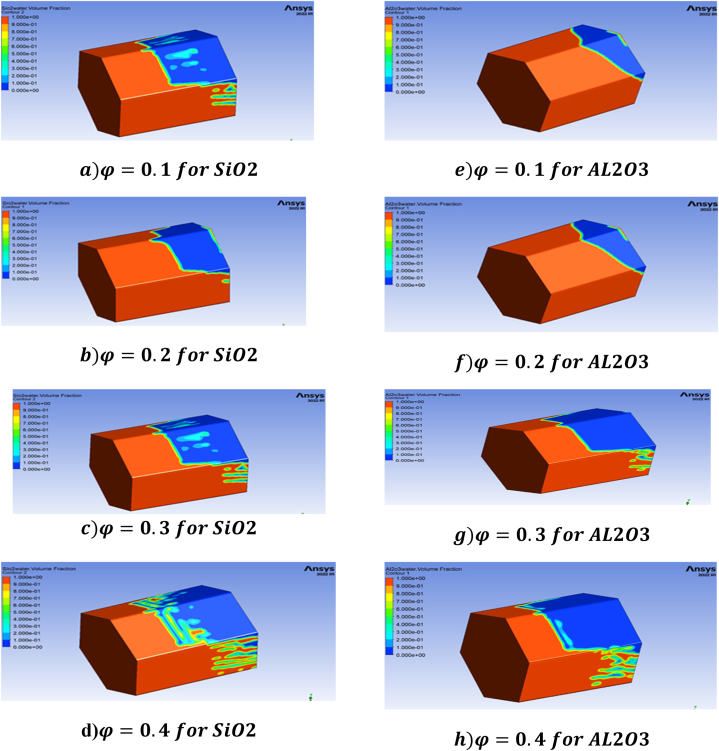
(a–d) represents effect of porosity on oil recovery using Sio2 and (e–h) represents porosity effect of Al2O3 nanoparticles on oil Recovery.

This research paper investigates the impact of a 3D porous hexagonal prism-shaped cavity to determine enhanced oil recovery by nanofluid injection. The ANSYS Fluent software simulates the partial differential equation system using the finite volume method (FVM). Porosity (0.1≤φ≤0.4), mass flow rate (0.05mL/min≤Q≤0.05ml/min), nanoparticle concentration (0.01≤ψ≤0.04), and the effect of relative permeability on oil and water saturation in the presence of gravity under different time durations are all investigated., Fig. 4(a–h) depicts the contour analysis of the impact of various porosity values i.e., (0.1≤φ≤0.4), on oil recovery rate over various time spans.

**Fig. 5:**
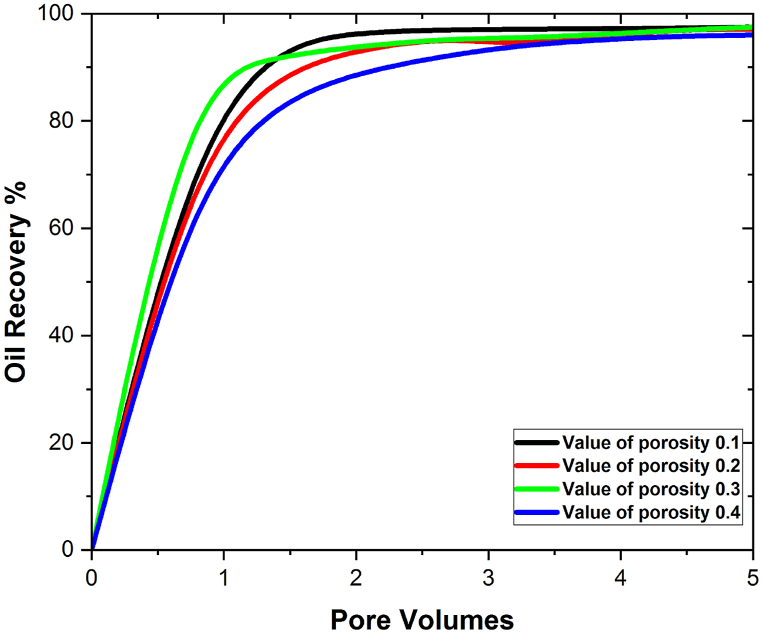
Effect of different values of porosity on oil recovery with SiO2 nanoparticles.

**Fig. 6:**
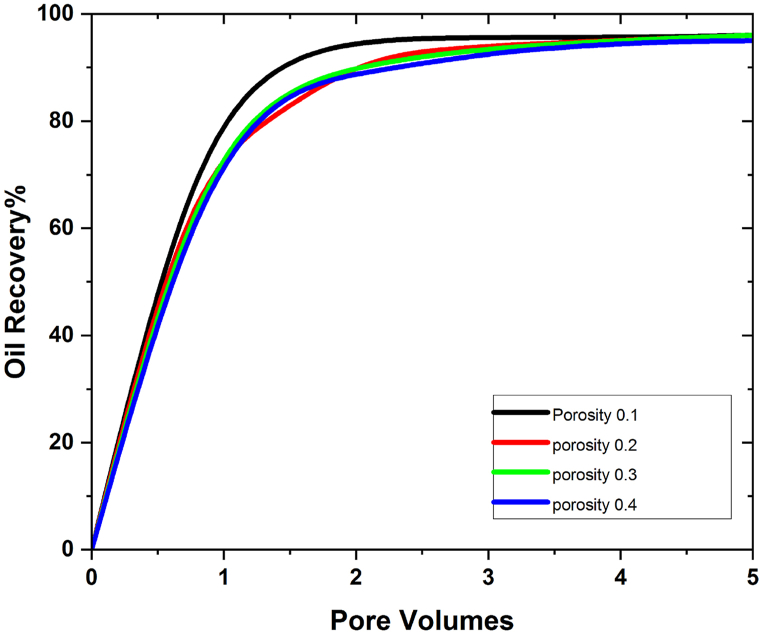
Effect of different values of porosity on oil recovery with Al2O3 nanoparticles.

Fig. 4(a–h) shows that the amount of oil recovered is directly related to the size of the 3D hexagonal porous prism cavity. Both the SiO2 and the Al2O3 scenarios exhibit this. Porosity is known to play a crucial role in the process of increasing the amount of oil that can be extracted from reservoirs [29]. We investigate the effect of porosity in different time spans to determine the maximum oil recovery rate, and it is discovered that increasing the time duration during the injection process enhances the rate of oil recovery at low values of porosity in the examined geometry. We investigated the influence of porosity on oil recovery rate to gain a better understanding. We looked at eight different pore volumes and discovered that the effect of porosity at 0.2, 0.3, and 0.4 on oil recovery rate is rather low at the beginning (i.e., the first two pore volumes), but that it grows in the subsequent pore volumes, reaching a maximum at pore volume 5. At this time, the greatest oil recovery for SiO2 nanoparticles is 98%, but the maximum oil recovery for Al2O3 is 95%, which is 3% less than the maximum oil recovery achieved with SiO2 nanoparticles. Fig. 5, Fig. 6 depict a graphical examination of oil recovery in relation to pore volumes for SiO2 and Al2O3.

**Fig. 7:**
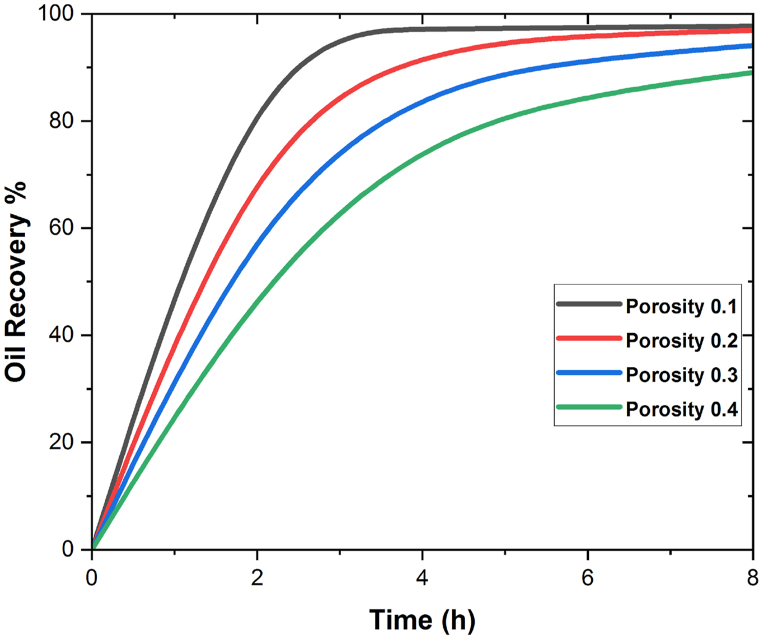
Effect of different values of porosity on oil recovery w. r.t time in SiO2.

**Fig. 8:**
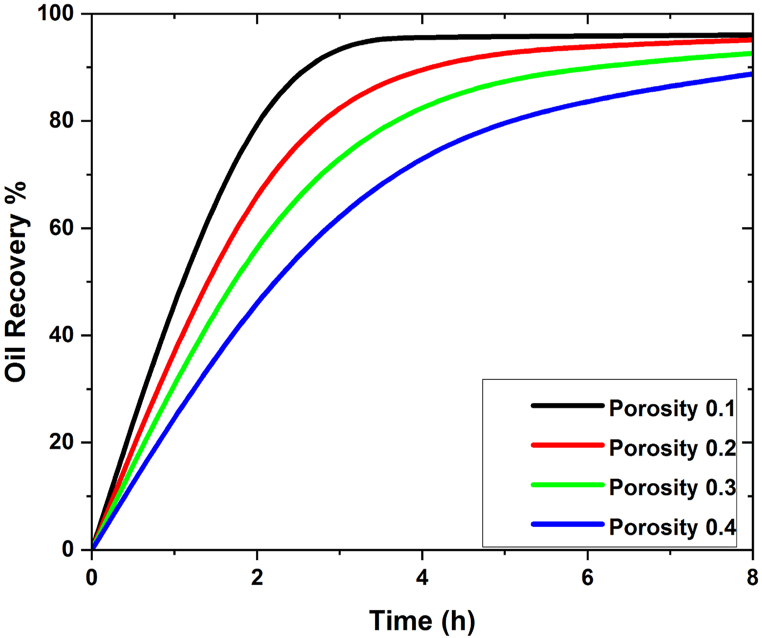
Effect of different values of porosity on oil recovery w. r.t time in Al2O3.

In Fig. 7, Fig. 8, the oil recovery rate for SiO2 and Al2O3 on different values of the porosity is shown numerically. It is also observed that for both cases i.e., in SiO2 and Al2O3 the maximum oil recovery is attained at 0.1. The effect of different porosity values on oil recovery rate is made clearer by the geometry of the reservoir and the flow parameters [38,39] In our 3D geometry, the most oil is recovered when the porosity is low. Improving how wet the medium is can help find the positive effect that nanoparticles have on the flooding process [39,40]. By strengthening this property, as well as modifying the viscosity of the injected fluid and raising the nanoparticle concentration to augment the Brownian diffusion force, the performance of the nanofluid flooding EOR process will be improved. In Fig. 7, Fig. 8, the impact of SiO2 and Al2O3 nanoparticles on oil recovery is graphically represented for the stated porosity parameters.

**Fig. 9:**
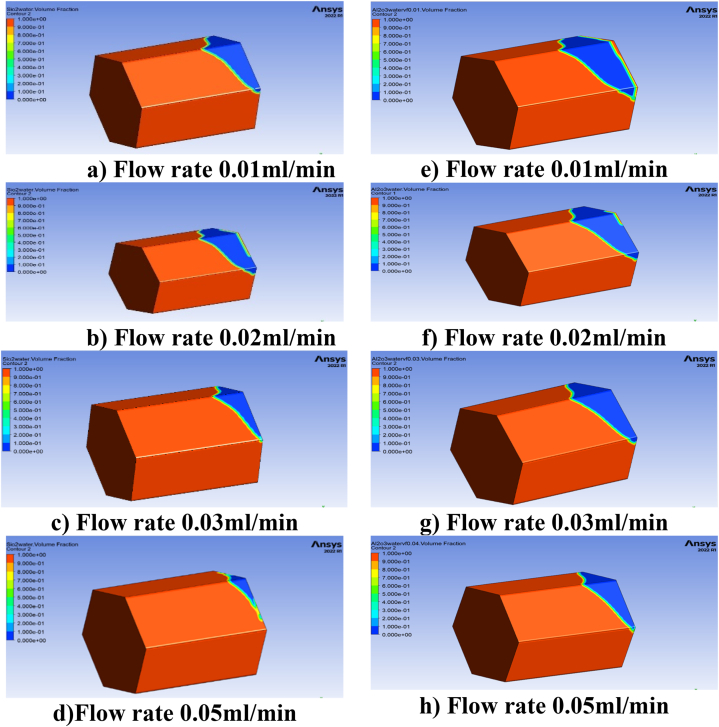
(a–d) Effect of flow rate different values of porosity on oil recovery for SiO2, (e–h) Effect of flow rate different values of porosity on oil recovery for Al2O3.

In the presence of silica and aluminium nanoparticles, it is also discovered that including the time parameter in the simulation process has a positive impact on the oil recovery rate. As time goes on, so does the rate of oil recovery, and the maximum oil recovery rate is reached after the 8-h simulations. It is also observed that when porosity decreases the oil recovery increase in both SiO2 and Al2O3 cases. The maximum oil recovery for SiO2 at final pore volume is 98% which is at 0.1 porosity. It is also important to explain that when the values of porosities decrease the oil recovery decreases due to the nature of the reservoir geometry. Moreover, it has been found that at 0.1, the greatest oil recovery rate is reached. Fig. 9(a–h) shows the contour analysis of mass flow rates on oil recovery at 0, 1, 2, 3, and 0.05 mL/min in the presence of silicon and aluminium nanoparticles.

**Fig. 10:**
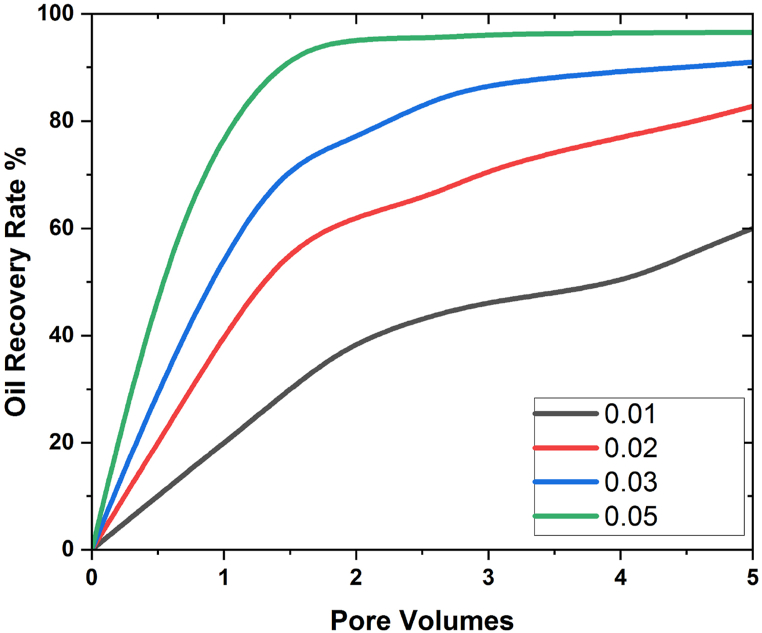
Analysis of Pore Volume versus oil recovery using SiO2.

**Fig. 11:**
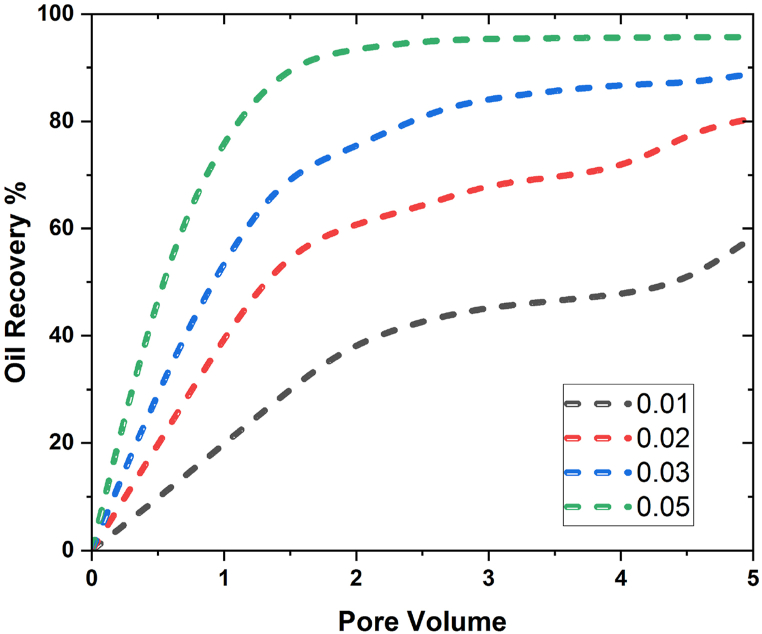
Analysis of Pore Volume versus oil recovery using Al203.

Dinesh et al. [41] also found that the flow rate has a positive effect on the amount of oil that can be recovered in the same case. Flow rate has the same effect on oil recovery when silicon and nanoparticles are present, which makes the filling process go faster. Fig. 10, Fig. 11 are representations that show additional details about how flow rate affects oil recovery.

**Fig. 12:**
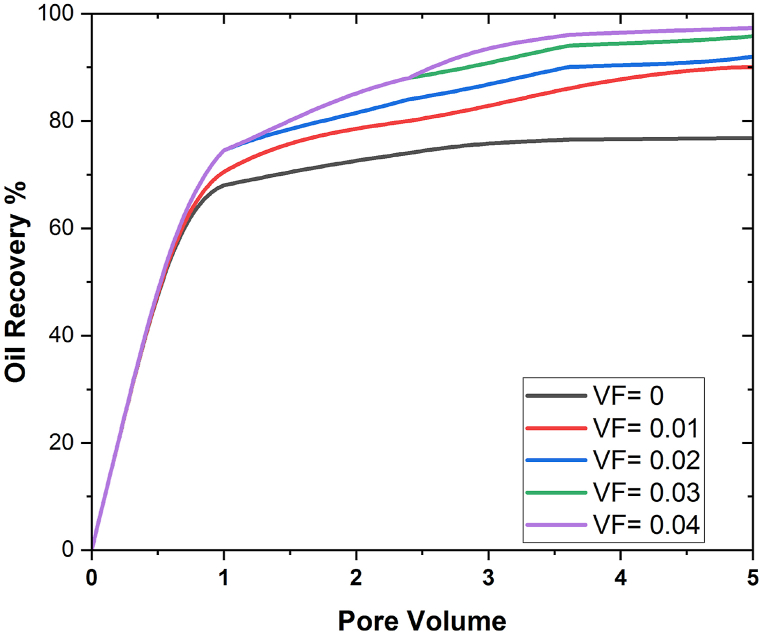
Influence of nanoparticle concentrations on oil recovery using AL203.

**Fig. 13:**
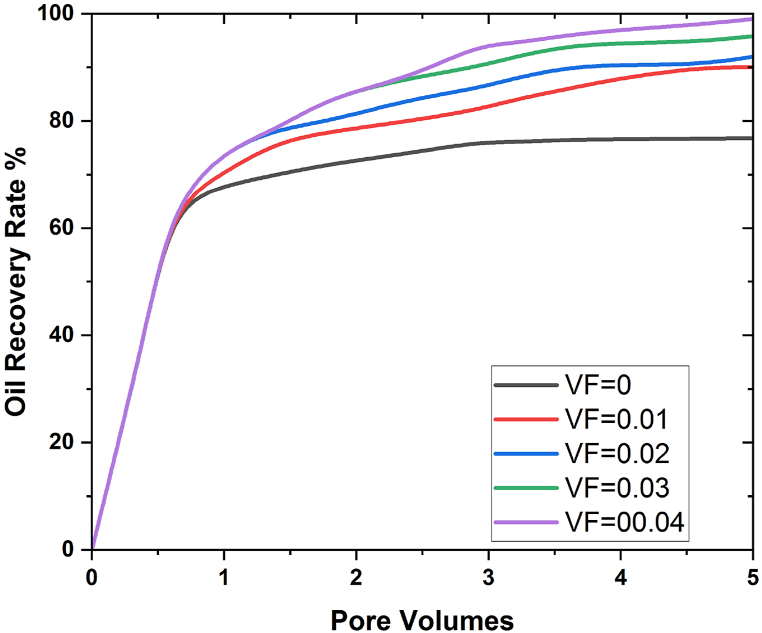
Effect of VF on oil recovery using SiO2.

In Fig. 10, Fig. 11, the effect of flow rate on eight pore volume is discussed, and it is found that oil recovery increases as flow rate going down, and the same thing was achieved when we increased the time of the simulation during the flooding process. At flow rate 0.01 for aluminium, the maximum oil recovery is 58%, and for silica nanoparticles, it is 62%; at flow rate 0.02 for aluminium 203, it is 80%, and with SiO2, it is 83%; at flow rate 0.03 for aluminium 203, and at flow rate 0.05, the maximum oil extraction is 95%, and for silicon nanoparticles, it is 97.5%. Consequently, considering the aforementioned findings, it is seen that when the flow rate decline, the oil recovery rises. This is because the increase in contact time that results from the flow rate decrease leads to the maximum interaction of molecules, which has a positive impact on oil recovery and, as a result, leads to the recovery of more oil. How the quantity of nanoparticles impacts the rate of oil recovery is one area that requires research. This is done in order to compare the effectiveness of the nanofluid flooding EOR method to that of the water flooding procedure. To determine how well the EOR process functions when nanofluid flooding is used, this is one of the issues that needs more research. Five different VFs, ranging from 0.01 to 0.05, were applied. Except for VF, all other factors were constant over the course of the experiment. At each stage, it was presumed that they were all the same. Fig. 12, Fig. 13 uses Al203 and SiO2 nanoparticles to display the oil recovery rate per volume of the injected pore at various VFs.

**Fig. 14:**
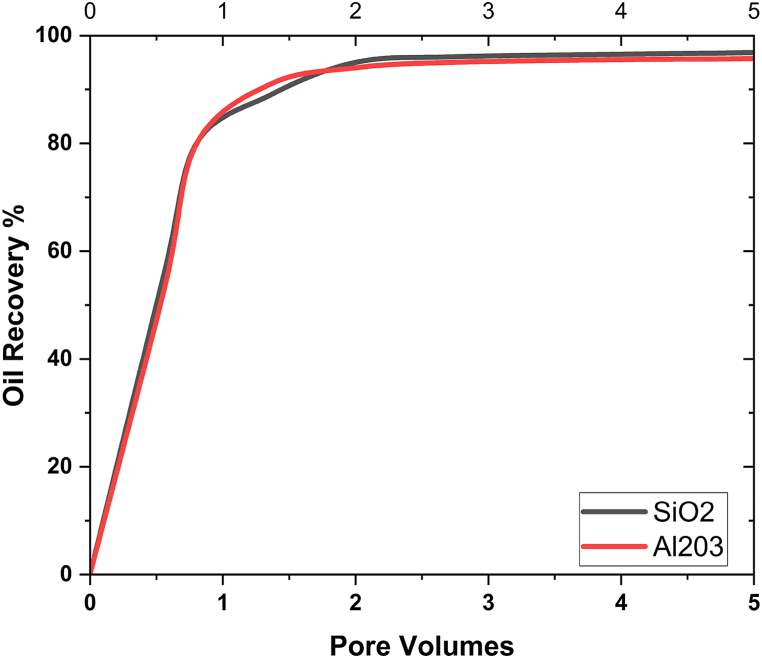
Effect of nanoparticles on oil recovery at different pore volumes.

This graph shows that when nanofluids (Ψ = 0) are used instead of pure water, more oil is recovered. The consequences also showed that oil could be removed from the 3D porous cavity more easily when the VF of the nanoparticles was increased. The medium's properties, specifically how well it wets, can be improved by using nanoparticles in the flooding process. The relative permeability of the two fluids is something else that must be considered in EOR and the solution of two-phase flows. In the study, adding nanoparticles to the water changed the way oil fluid and water pass through each other. Fig. 14 illustrates the main factors that contributed to EOR. It demonstrates how the relative permeability of nanofluid increased while the permeability of oil decreased as a result of the presence of nanoparticles. The relative permeability of the water was increased as a result of this operation, which enhanced the flow of the oil. In conclusion, employing nanofluids instead of water considerably increased the EOR process' performance. Literature has also reported similar results [[42], [43], [44]]. Geometry is used in nanoflooding for oil recovery by considering the size and form of nanoparticles, understanding the geometry of porous media, optimizing injection patterns and well placement, and analyzing capillary and interfacial forces. By combining 3D complex geometry with effective nanoflooding technologies, oil recovery from reservoirs can be improved [1,11,45].

The effect of the oil recovery at different pore volumes for both nanoparticles is shown in Fig. 14. It is noticeable that the oil recovery rate increases when pore volume increases. The maximum oil recovery obtained at the final pore volume is 96.8% in the case of SiO2 and 94.5% in the case of Al2O3 nanoparticles, which shows that in the case of SiO2, more oil recovery is achieved. It is since when pore volume is large, there is a strong possibility of increasing the flow motion, which increases the sweep efficiency and hence the maximum oil recovered.

## 4 Conclusions

In this paper, the numerical investigation of a three-dimensional hexagonal porous cavity is pursued to predict out the oil recovery by investigating different nanoparticles. To find out the oil recovery, the parameters of volume fraction, porosity, flow rate, and relative permeability are studied. Based on the results, the following are the conclusions and remarks:

• The maximum oil recovery is attained at low values of mass flow rate in the 3D hexagonal prism in the presence of silicon and aluminium nanoparticles.
• The effect of porosity has a positive impact on the flooding process, and at low porosity values, the recovery rate is very high.
• The VF of nanoparticles increases the oil recovery rate, and the maximum oil recovery in SiO2 nanoparticles is 99%. In our problem, the most oil can be extracted from a rock with low porosity and a high concentration of nanoparticles.
• It is also observed that the use of SiO2 gives a better oil recovery rate than Al2O3.

## Author contribution statement

Mudasar Zafar: Conceived and designed the experiments; Performed the experiments; Analyzed and interpreted the data; Contributed reagents, materials, analysis tools or data; Wrote the paper.

Hamzah Sakidin: Conceived and designed the experiments; Performed the experiments; Contributed reagents, materials, analysis tools or data.

Mikhail Sheremet: Performed the experiments; Contributed reagents, materials, analysis tools or data.

Iskandar Dzulkarnain: Analyzed and interpreted the data; Contributed reagents, materials, analysis tools or data; Wrote the paper.

Roslinda Nazar: Performed the experiments; Analyzed and interpreted the data.

Abdullah Al-Yaari: Conceived and designed the experiments; Analyzed and interpreted the data.

Nur Asyatumaila Mohamad Asri: Performed the experiments; Analyzed and interpreted the data; Wrote the paper.

Mohd Zuki Salleh: Performed the experiments; Analyzed and interpreted the data; Contributed reagents, materials, analysis tools or data.

Shazia Bashir: Performed the experiments; Contributed reagents, materials, analysis tools or data; Wrote the paper.

## Data availability statement

No data was used for the research described in the article.

## Declaration of competing interest

The authors declare that they have no known competing financial interests or personal relationships that could have appeared to influence the work reported in this paper.

## Nomenclature

Bc: Pressure Parameter
D: Diffusion coefficient
k: Permeability
$K_{p}$: Pore throat blocking
$K_{d}$: surface retention rate of nanoparticlesp Pressure
R: Retention term of nanoparticles
S: Saturation term
t: Time
u: Fluid velocity
VF: Nanoparticle Volume Fraction

**Greek Letters**
β: Bulk surface area
∇: Gradient operator
ρ: Density
μ: Viscosity
φ: Porosity
ψ: Nanoparticle concentrations (Volumetric Concentration)
o, o*: volume of the nanoparticles in porous media
υ: Critical velocity of the nanoparticles to enter in media
Ψ: Surface area of the porous medium

**Subscript**
abs: Absolute
c: Capillary pressure
d: Desorption
e: Effectiveness
g: Rock grain size
o: Oil
p: Diameter of nanoparticles
ro: Oil residual
rw: Water residual
rw, p: Relative permeability of water
rw, o: Relative permeability of oil
w: Water
